# Supplementary material for: PM2.5 concentration assessment based on geographical and temporal weighted regression model and MCD19A2 from 2015 to 2020 in Xinjiang, China
Source: PLoS One. 2023 May 11;18(5):e0285610. doi: 10.1371/journal.pone.0285610 (PMC10174561; doi:10.1371/journal.pone.0285610)
Supplement: S1 File — (DOCX) [file pone.0285610.s001.docx]

PM_2.5_ concentration assessment based on geographical and temporal weighted regression model and MCD19A2 from 2015 to 2020 in Xinjiang, China

Weilin Quan ^1,2,3¶^ Nan Xia ^1,2,3*¶^ ,Yitu Guo ^1,2,3^ ,Wenyue Hai^1,2,3^,Jimi Song^1,2,3^ and Bowen Zhang^1,2,3^

^1^ College of Geography and Remote Sensing Sciences, Xinjiang University, Urumqi 830046, China

^2^ Xinjiang Key Laboratory of Oasis Ecology, Xinjiang University, Urumqi 830046, China

^3^ Key Laboratory of Smart City and Environment Modelling of Higher Education Institute, Xinjiang University, Urumqi 830046, China

***** Correspondence author

E-mail: [xn_gis@xju.edu.cn](mailto:xn_gis@xju.edu.cn)

^¶^ These authors contributed equally to this work.

**CONTAINS**

**Figure S1. Monthly mean heat map of PM_2.5_ concentration from 2015 to 2020**

**Table S1. Statistical results of annual PM_2.5_ concentration in Xinjiang**

**Table S2. Statistical results of seasonal PM_2.5_ concentration in Xinjiang**

**Fig S1. Monthly mean heat map of PM_2.5_ concentration from 2015 to 2020**


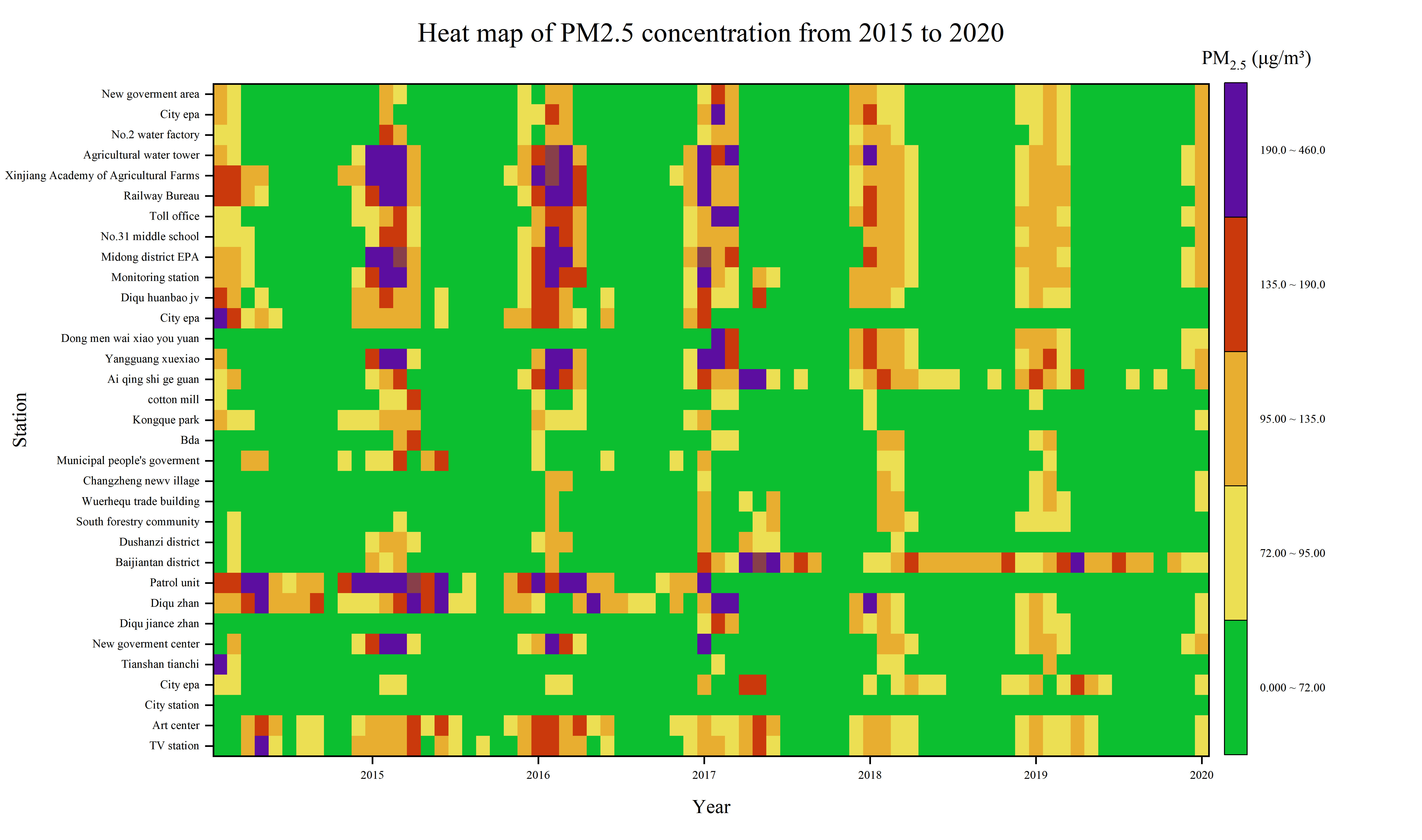


**Table S1. Statistical results of annual PM2.5 concentration in Xinjiang**

| **Area** | **2015**  **(μg/m³)** | **2016**  **(μg/m³)** | **2017**  **(μg/m³)** | **2018**  **(μg/m³)** | **2019**  **(μg/m³)** | **2020**  **(μg/m³)** |
| --- | --- | --- | --- | --- | --- | --- |
| Urumqi | 55.41 | 73.50 | 56.27 | 39.75 | 37.72 | 34.76 |
| Karamay City | 43.85 | 75.87 | 54.82 | 42.46 | 46.23 | 35.35 |
| Turpan City | 69.15 | 75.33 | 62.03 | 63.33 | 40.26 | 32.52 |
| Hami City | 56.52 | 83.94 | 43.93 | 44.62 | 37.45 | 29.46 |
| Changji Hui Autonomous Prefecture | 51.86 | 77.27 | 62.55 | 43.40 | 44.27 | 35.11 |
| Bortala Mongolian Autonomous Prefecture | 60.69 | 59.03 | 37.31 | 34.90 | 35.09 | 21.54 |
| Bayingolin Mongolian Autonomous Prefecture | 80.01 | 93.20 | 70.64 | 73.34 | 50.58 | 35.72 |
| Aksu Region | 89.24 | 93.82 | 78.50 | 67.87 | 53.82 | 44.07 |
| Kizilsu Kirgiz Autonomous Prefecture | 51.72 | 79.06 | 43.35 | 50.23 | 39.78 | 27.03 |
| Kashgar region | 69.01 | 85.62 | 78.96 | 50.88 | 44.07 | 40.11 |
| Hotan District | 78.61 | 101.42 | 85.85 | 64.71 | 61.16 | 46.62 |
| Ili Kazakh autonomy | 64.73 | 40.53 | 34.23 | 33.91 | 27.30 | 19.24 |
| Tacheng area | 46.17 | 65.82 | 44.37 | 42.00 | 44.08 | 31.49 |
| Altay Region | 40.07 | 69.90 | 46.12 | 40.69 | 46.27 | 32.96 |
| Shihezi City | 48.59 | 69.70 | 62.25 | 61.71 | 60.69 | 46.14 |
| Wujiaqu City | 57.40 | 83.04 | 81.30 | 62.54 | 65.18 | 53.37 |
| Alar City | 110.73 | 75.66 | 91.13 | 66.41 | 58.72 | 50.36 |
| Tumushuk City | 90.30 | 96.95 | 96.73 | 57.11 | 52.97 | 44.97 |

**Table S2. Statistical results of seasonal PM_2.5_ concentration in Xinjiang**

| **Area** | **Spring**  **(μg/m³)** | **Summer**  **(μg/m³)** | **Autumn**  **(μg/m³)** | **Winter**  **(μg/m³)** |
| --- | --- | --- | --- | --- |
| Hotan District | 60.48 | 91.36 | 42.80 | 95.84 |
| Kizilsu Kirgiz Autonomous Prefecture | 40.68 | 20.68 | 34.67 | 56.10 |
| Kashgar region | 60.89 | 32.59 | 47.84 | 75.91 |
| Aksu Region | 81.13 | 53.49 | 55.22 | 76.71 |
| Bayingolin Mongolian Autonomous Prefecture | 57.92 | 56.25 | 42.66 | 65.08 |
| Turpan City | 43.16 | 25.3 | 36.57 | 82.26 |
| Changji Hui Autonomous Prefecture | 33.37 | 20.46 | 32.28 | 58.37 |
| Urumqi | 36.30 | 18.62 | 36.29 | 65.77 |
| Shihezi City | 30.82 | 29.49 | 41.15 | 65.56 |
| Wujiaqu City | 30.32 | 25.46 | 34.77 | 95.43 |
| Hami City | 37.51 | 22.89 | 32.75 | 84.07 |
| Ili Kazakh autonomy | 45.89 | 20.26 | 33.24 | 48.90 |
| Altay Region | 35.78 | 21.65 | 34.71 | 56.33 |
| Bortala Mongolian Autonomous Prefecture | 52.79 | 23.73 | 40.02 | 46.03 |
| Tacheng area | 36.62 | 22.8 | 33.70 | 47.01 |
| Karamay City | 40.27 | 22.65 | 35.26 | 37.27 |
| Alar City | 111.02 | 65.91 | 79.79 | 74.38 |
| Tumushuk City | 105.15 | 68.99 | 72.94 | 77.64 |
