# Supplementary material for: PM2.5 concentration assessment based on geographical and temporal weighted regression model and MCD19A2 from 2015 to 2020 in Xinjiang, China
Source: PLoS One. 2023 May 11;18(5):e0285610. doi: 10.1371/journal.pone.0285610 (PMC10174561; doi:10.1371/journal.pone.0285610)
Supplement: Attachment — Submitted filename: Comments-PONE-D-22-28837.pdf [file pone.0285610.s002.pdf]

“PM2.5 concentration assessment based on geographical and temporal weighted regression model and MCD19A2 from 2015 to 2020 in Xinjinag, China”

High quality and spatial resolution of PM2.5 data is necessary for assessing the air quality and public health. This study proposes an interesting method for deriving the PM2.5 datasets with high quality and spatial resolution. Considering several issues, I recommend for potential publication after Minor revision.

Specific comments:
Title: the authors should clearly state at what temporal scale, which would be better for readers.

Introduction: Line 75-85, the review of $PM_{2.5}$ retrieval models is insufficient. Though GTWR is a promising method for $PM_{2.5}$ estimations, recently, some machine learning and deep learning models can also consider spatiotemporal correlation as well as have a higher estimation accuracy. Therefore, the author need to do a further review of $PM_{2.5}$ retrieval models and talk more about why the GTWR model was chosen for $PM_{2.5}$ retrievals. For instance, Predicting annual PM 2.5 in mainland China from 2014 to 2020 using multi temporal satellite product: An improved deep learning approach with spatial generalization ability, *ISPRS Journal of Photogrammetry and Remote Sensing*.

Method: The authors should give a flowchart on more clearly describing the main method. One issue makes me confusing that the GWR is proposed for considering land surface characteristics, while PM2.5 is a non-normal distribution meteorological variable. So the suitability of GWR family for predicting PM 2.5 needs to be further discussed. While for downscaling satellite-based precipitation products, land surface characteristics were generally utilized using GWR method, for instance, A spatial data mining algorithm for downscaling TMPA 3B43 V7 data over the Qinghai–Tibet Plateau with the effects of systematic anomalies removed, *Remote Sensing of Environment*.

Figures: The quality of all the Figures should be further improved for meeting the publication standard.
