# Supplementary material for: PM2.5 concentration assessment based on geographical and temporal weighted regression model and MCD19A2 from 2015 to 2020 in Xinjiang, China
Source: PLoS One. 2023 May 11;18(5):e0285610. doi: 10.1371/journal.pone.0285610 (PMC10174561; doi:10.1371/journal.pone.0285610)
Supplement: Attachment — Submitted filename: Response to Reviewers.docx [file pone.0285610.s003.docx]

**Responses to reviewer’s comments**

NOTE: **RC**=Reviewer’s comment; **AR**=Authors’ response

Reviewer(s) Comments:

**Reviewer #1**

*High quality and spatial resolution of PM_2.5_ data is necessary for assessing the air quality and public health. This study proposes an interesting method for deriving the PM_2.5_ datasets with high quality and spatial resolution. Considering several issues, I recommend for potential publication after Minor revision.*

**AR:**Thank you very much for your kindly comments on our manuscript. There is no doubt that these comments are valuable and very helpful for revising and improving our manuscript. In what follows, we would like to answer the questions you mentioned and give detailed account of the changes made to the original manuscript.

**RC:***[1]Title: the authors should clearly state at what temporal scale, which would be better for readers.*

**AR:**The title of this paper has been modified to:PM_2.5_ concentration assessment based on geographical and temporal weighted regression model and MCD19A2 from 2015 to 2020 in Xinjiang, China.We are very much appreciated for your constructive comments and insightful suggestion.

**RC:***[2]Introduction: Line 75-85, the review of PM_2.5_ retrieval models is insufficient. Though GTWR is a promising method for PM_2.5_ estimations, recently, some machine learning and deep learning models can also consider spatiotemporal correlation as well as have a higher estimation accuracy. Therefore, the author need to do a further review of PM_2.5_ retrieval models and talk more about why the GTWR model was chosen for PM_2.5_ retrievals. For instance, Predicting annual PM _2.5_ in mainland China from 2014 to 2020 using multi temporal satellite product: An improved deep learning approach with spatial generalization ability, ISPRS Journal of Photogrammetry and Remote Sensing.*

**AR:**Thank you for the helpful suggestion.We have further reviewed the model of the PM_2.5_ inversion, and discussed the reasons for choosing GTWR model in "Introduction".

**RC:***[3]Method: The authors should give a flowchart on more clearly describing the main method. One issue makes me confusing that the GWR is proposed for considering land surface characteristics, while PM_2.5_ is a non-normal distribution meteorological variable. So the suitability of GWR family for predicting PM_2.5_ needs to be further discussed. While for downscaling satellite-based precipitation products, land surface characteristics were generally utilized using GWR method, for instance, A spatial data mining algorithm for downscaling TMPA 3B43 V7 data over the Qinghai–Tibet Plateau with the effects of systematic anomalies removed, Remote Sensing of Environment.*

**AR:**This is very helpful and insightful suggestion. Thank you so much.We have drawn a flow chart to more clearly describe the main methods (Figure 3).We think about the applicability of GWR prediction in PM_2.5_: First, in our experiments, GWR has good inversion accuracy (six year average R^2^=0.64, RMSE=23.71); Secondly, predecessors used GWR model to retrieve PM_2.5_ concentrations in some regions of China and obtained good results, such as Rui, etc. We have added a reference to this paper in the paper; Finally, we understand the distribution of PM_2.5_ in this way. Xinjiang covers a large area but has a small population. As a whole, PM_2.5_ is more affected by natural factors. When natural factors are the dominant factors, PM_2.5_ presents a normal distribution, while in some local areas, such as urban agglomerations on the northern slope of Tianshan Mountains, PM_2.5_ is mainly affected by human factors and presents a non normal distribution. Therefore, whether the existence of PM_2.5_ in Xinjiang is normal or non normal, It is worth further discussion and research. This is only our speculation based on our understanding of the overview of the study area, and it is only for the reviewers' criticism. Based on the above reasons, we chose the GWR model.

Figure 3. Workflow outline

**RC***:[4]Figures: The quality of all the Figures should be further improved for meeting the publication standard.*

**AR:**Thank you very much!Thank you.We have increased the resolution of each figure in the paper to 300dpi, and tested the quality of each figure in PACE. There is no ambiguity of the figure. However, after uploading figures and generating PDF files in the submission system, the resolution of each figures in the PDF file will be reduced. Please click the picture download link in the upper right corner of the PDF file to obtain high-resolution and high-quality figures.

NOTE: **RC** = Reviewer’s comment; **AR** = Authors’ response

Reviewer(s) Comments:

**Reviewer #2**

*I'm finding many problems with the grammar and organization that would lead me to suggest that it still needs editing by someone with some subject area experience. While the topic is good and the analysis is well-done, the importance of the research needs to be better explained.*

**AR:**Thank you very much for your useful comments on our manuscript .We are very sorry for the mistakes in this manuscript and inconvenience they caused in your reading. The manuscript has been thoroughly revised and edited by a native speaker, so we hope it can meet the journal’s standard.

**RC***:[1]Section Methods. It makes no sense to describe in detail each of the methods. I recommend that the authors omit all the formulas and describe each of their methods more superficially.*

**AR:**Thank you very much for your valuable suggestions! According to your suggestion, we have briefly described each method, but we think that the formula is necessary, so we have omitted the formula as much as possible, and only the main part has been retained.

**RC***:[2]The article lacks a critical analysis of the change in PM2.5 and AOD. Pay more attention to the actual variation of these pollutants in different areas of the province. It would be helpful if the authors focused more on dust aerosols and the impact of dust storms. Enough papers have already been published on this topic, so the authors can cite one of published articles.*

**AR:**Thank you for the helpful suggestion.Based on your suggestions, we have supplemented the critical analysis of changes in PM_2.5_ and AOD[now in lines 281-288], and cited the following papers at appropriate locations:

[1].Liu, J., Ding, J., Rexiding, M., Li, X., Zhang, J., Ran, S., Bao, Q., & Ge, X. (2021). Characteristics of dust aerosols and identification of dust sources in Xinjiang, China. Atmospheric Environment, 262, 118651. [now in lines 349]

**RC***:[3]Why don't the authors mention the accuracy of MODIS products. Moreover, there are many studies on the mutual verification of MODIS and AERONET (Bilal et al., 2019; Filonchyk and Hurynovich, 2020).*

**AR:**Thank you very much! According to your suggestion, we have supplemented the description of data accuracy in "Data and Method" and cited the following papers:

[1]. Filonchyk, M., & Hurynovich, V. (2020). Validation of MODIS aerosol products with AERONET measurements of different land cover types in areas over Eastern Europe and China. Journal of Geovisualization and Spatial Analysis, 4(1), 1-11.
[2]. Bilal, M., Nazeer, M., Nichol, J., Qiu, Z., Wang, L., Bleiweiss, M. P., ... & Lolli, S. (2019). Evaluation of Terra-MODIS C6 and C6. 1 aerosol products against Beijing, XiangHe, and Xinglong AERONET sites in China during 2004-2014. Remote Sensing, 11(5), 486. [now in lines 125]

**RC***:[4]"Fig 2. AOD interpolation diagram from 2015-2020". Looking at Figure 2, I noticed that it has nothing to do with the diagram. As I see it, this is the spatial distribution of the AOD.*

**AR:**Thank you. We have changed the name of the figure from"Fig 2. AOD interpolation diagram from 2015-2020" to "Fig 2. Spatial distribution map of AOD in Xinjiang from 2015-2020"

**RC***:[5]Why don't authors use the same color scheme for all figures?*

**AR:**Thank you.Since Figure 2 represents the distribution of AOD, and Figure 5 and Figure 6(now Figure 6 and Figure 7) represent the distribution of PM_2.5_, their attributes are different, so we use different colors to distinguish them. For Figure 9(now Fogure 10), if the same color is used, the boundary of the area in Figure 9 will be blurry and difficult to distinguish, because the same color band represents the same value range, while the maximum and minimum values of PM_2.5_ in four seasons are different, and the same color represents different value ranges, which will lead to no comparability. For example, the maximum value in summer is 270.34μg/m^3^, the maximum value in autumn is 157.74μg/m^3^, when using the same color band, the values represented by the same color are different. Therefore, in consideration of the aesthetics of the drawing and the distinguishability of the pictures, we chose different colors for drawing.

NOTE: **RC**=Reviewer’s comment; **AR**=Authors’ response

Reviewer(s) Comments:

**Reviewer #3**

*This manuscript compares several model’s performance in retrieving PM_2.5_,and give a spatial and temporal patten of PM_2.5_, it would be better if the research content go a little deeper. Some problems need to be solved:*

**AR:**We feel great thanks for your professional review work on our article. According to your nice suggestions, we have made extensive corrections to our previous draft, the detailed corrections are listed below:

**RC***:[1] In line 171, you did’t give the mathematical notation for the humidity influence factor.*

**AR:**Thank you for pointing this out. We added the symbol of humidity influencing factor in line 171.[now in lines 165]

**RC***:[2] In line 186, the symbol for the random variable disappeared.*

**AR:**Thank you for pointing this out.We added the symbol of the random variable in line 186.[now in lines 175]

**RC***:[3] Table 3 needs to be adjusted to be more aesthetically pleasing.*

**AR:**Thank you for the helpful suggestion.We have adjusted Table 3.

**RC***:[4] In line 248, the comma was in a wrong place.*

**AR:**Thank you for pointing this out.We have corrected the use of 248 lines of commas.[now in lines 219]

**RC***:[5] Does Figure 4 use multi-year average data for analysis? It is not clearly marked.*

**AR:**Thank you.Figure 4 (now Figure 5) is analyzed using the six-year average data of each season. According to your suggestion, we have added a description of the data used in Figure 4.[now in lines 244-245]

**RC***:[6] The sentence in line 315, it’s unclear for understanding.*

**AR:**Thank you very much！We adjusted the 315 line statement to make it easier to understand.[now in lines 304-305]

**RC***:[7] The discussion needs to be improved.*

**AR:**Thanks for your valuable counsel.The discussion section has been improved and incorporated into "Results and Discussion"

NOTE: **RC**=Reviewer’s comment; **AR**=Authors’ response

Reviewer(s) Comments:

**Reviewer #4**

*In this study, the authors applied the SLR, GWR, and GTWR models with MCD19A2, NDVI, DEM, and meteorological data to retrieve PM_2.5_ concentrations from year 2015 to 2020 in Xinjiang. The performance of each model is compared, the optimal model (GTWR) is selected for PM_2.5_ inversion. The spatiotemporal distribution characteristics of PM_2.5_ and meteorological impact factors are further analyzed. This topic is interesting; however, the manuscript is not well organized and the writing of the paper is not good, which makes the manuscript looking more like a project report instead of a scientific research paper. I thinks the paper needs a lot of work to be improved.*

**AR:**Thank you very much for your kindly comments on our manuscript. These comments are all valuable and very helpful for revising and improving our paper, as well as the important guiding significance to our researches. We have studied comments carefully and have made corrections：

**RC***:[1] All abbreviations appearing at the first time (including abstract) should be given full expression.*

**AR:**Thank you for pointing this out.We have supplemented the full description of all abbreviations appearing for the first time in the text.include:

(1) MCD19A2 (MODIS/Terra+Aqua Land Aerosol Optical Thickness Daily L2G Global 1km SIN Grid V006)
(2) MODIS (Moderate-resolution Imaging Spectroradiometer)
(3) MOD04_L2 (MODIS Terra/Aqua Aerosol 5-Min L2 Swath 10km)
(4) MOD04_3K (MODIS Terra/Aqua Aerosol 5-Min L2 Swath 3km)
(5) MAIAC (Multi-Angle Implementation of Atmospheric Correction)
(6) National Aeronautics and Space Administration (NASA)
(7) European Centre for Medium-Range Weather Forecasts (ECMWF)
(8) Akaike information criterion（AICc)

**RC***:[2]What is the definition of “small- and medium-size area” ? Is Xijiang (with area of 166×104 km2) a small- and medium-size area? I cannot agree with this subjective description.*

**AR:**Thank you for your comments. We corrected the subjective description of "small and medium-sized areas" in the text.

**RC***:[3]Line 93-Line107. This paragraph looks like a translation from a project report, which is irrelevant to the major research aim of this paper. Readers cannot get any implications from this paragraph why Xijiang is selected.*

**AR:**Thank you very much for your constructive comments and suggestions! We have revised lines 93-107, focusing on the reasons for choosing Xinjiang.

[now in lines 91-107]

**RC***:[4]Line 109: It is better to change “Materials and Methods” to “Data and Method”.*

**AR:**The "Materials and Methods" has been revised to “Data and Method”.Thank you again.[now in lines 109]

**RC***:[5] Line 114-115 “After excluding the sites with many missing monitoring data and many invalid values”, this description seems very casual. How many sites are in the original dataset? What principle is used to conduct the data quality control? How many sites are deleted?*

**AR:**Thank you for pointing this out.Our original data includes 35 stations in total. Since the city environmental monitoring station in Kashgar region (in the southwest of Xinjiang) has four months of missing PM_2.5_ data, and the other monitoring station WuBan , which has 73 months of missing or invalid data, we deleted these two stations. In the data quality control, we adopt the principle that the PM_2.5_ value remains unchanged for more than 12 consecutive hours, the hourly concentration value deviates by more than 3 times the standard deviation of the 24 hour mean value of the mass concentration of the day, and the mass concentration value is greater than 999μg/m^3^ as invalid values. Cite line xx in the form of reference.[now in lines 113-116]

**RC***:[6]Line 116-118, the authors mentioned that “To match the AOD data in time and space, the average PM2.5 value of each station one hour before and after the satellite transit is calculated to determine the daily, monthly, quarterly, and annual averages of each station”. Using 1-2 hours data to determine the daily, monthly, quarterly and annual averages will raise large uncertainties.*

**AR:**Thanks for your valuable counsel.There is only one MCD19A2 data every day. We need to match the ground monitoring data according to the satellite transit time. This uncertainty cannot be avoided at present, so we have minimized this uncertainty.

**RC***:[7]Why do the authors only download AOD data for 1st, 15th and 30th of each month?*

**AR:**Thank you for your valuable comments. Using the mean value of AOD data at the beginning, middle and end of a month to replace the monthly mean value will lead to some uncertainties. Ideally, we should use daily data for the experiment, but the amount of data will be very large, which is not conducive to the experiment. In addition, we focus more on the feasibility of model inversion of PM_2.5_ in our research. Later, we can use the AOD dataset of complete time series to synthesize PM_2.5_ products, so as to improve the accuracy of data.

**RC***:[8]Line 171, which parameter represents the humidity influence factor? “f(RH)” is missing.*

**AR:**Thank you for pointing this out.We have added the missing "f (RH)" in line 171.[now in lines 165]

**RC***:[9]Line 186, which is a random variable?*

**AR:**In line 186, "$\varepsilon$" represents a random variable. We have added it to this line. Thank you again for your valuable comments.[now in lines 175]

**RC***:[10]“Results” and “Discussion” can be combines to Section “Results and Discussions”.*

**AR:**Thanks for your valuable counsel.We have moved the discussion to the results section and merged them into "results and discussion".

**RC***:[11]What is “AICc”? there is no definition at all.*

**AR:**Thank you for pointing this out.We have supplemented the definition of AICc in "Model Comparison".[now in lines 219-221]

**RC***:[12]Section “PM2.5 Year Distribution in Xinjing”, this section uses lots of words to describe the phenomenon, without any further explanations.*

**AR:**Thank you for the helpful suggestion,We have further explained and discussed this phenomenon in the section "Annual distribution of PM_2.5_ in Xinjiang", and specifically analyzed PM_2.5_ with DEM and official data [now in lines 281-288].

**RC***:[13]Suggest to move “discussions” to “results” and combine to one Section “Results and Discussions”*

**AR:**Thanks for your valuable counsel.We have moved the discussion to the results section and merged them into "results and discussion".

**RC***:[14]Conclusion: the 3 paragraphs seems strange, please combine the three paragraphs and conclude the major findings of this study.*

**AR:**Thank you for the helpful suggestion. We further summarize the main findings of this study.

**RC***:[15]There are quite a lot of grammar errors or typos, the language should be polished thoroughly.*

**AR:**Thank you very much for your useful comments on our manuscript .We are very sorry for the mistakes in this manuscript and inconvenience they caused in your reading. The manuscript has been thoroughly revised and edited by a native speaker, so we hope it can meet the journal’s standard.
