# Supplementary material for: PM2.5 concentration assessment based on geographical and temporal weighted regression model and MCD19A2 from 2015 to 2020 in Xinjiang, China
Source: PLoS One. 2023 May 11;18(5):e0285610. doi: 10.1371/journal.pone.0285610 (PMC10174561; doi:10.1371/journal.pone.0285610)
Supplement: Attachment — Submitted filename: Responses to reviewer.docx [file pone.0285610.s004.docx]

**Responses to reviewer’s comments**

NOTE: **RC**=Reviewer’s comment; **AR**=Authors’ response

Reviewer(s) Comments:

**Reviewer #2**

*The authors have done a good job of improving their manuscript. Therefore, I recommend this article and hope that this article will contribute to the scientific community.*

**AR:**Thank you very much for your kind work and recognition of our manuscript！Your suggestions have been very helpful in modifying and improving our manuscript.

NOTE: **RC** = Reviewer’s comment; **AR** = Authors’ response

Reviewer(s) Comments:

**Reviewer #4**

*The authors have carefully addressed the reviewers’ comments and the quality of the manuscript has been substantially improved. However, my major concern is that this study only used AOD data from the first, 15th, and 30th of each month in Xinjiang from 2015 to 2020. In this case, the results describing trends of PM2.5 will raise large uncertainties. At least detailed comparisons between your retrieved monthly/yearly average results at specific grids (covering the surface sites) and the ground observations covering the long time period should be given. The uncertainties should be carefully discussed. In addition, there are some minor typos that need to be revised.*

**AR:**Thank you very much for your kindly comments on our manuscript. There is no doubt that these comments are valuable and very helpful for revising and improving our manuscript. In what follows, we would like to answer the questions you mentioned and give detailed account of the changes made to the original manuscript.

**RC***:[1]At least detailed comparisons between your retrieved monthly/yearly average results at specific grids (covering the surface sites) and the ground observations covering the long time period should be given. The uncertainties should be carefully discussed.*

**AR:**Thank you very much for your valuable suggestions.We compared the monthly/yearly average results at specific grids (covering the surface sites) with the ground observation results covering a long period of time , and discussed the uncertainties in the Data uncertainty analysis. [now in lines 404-422]

**RC***:[2]Line 110 “Data” can be deleted.*

**AR:**Thank you very much! We have deleted "DATA" on line 110.

**RC***:[3] Line 298, two “Fig7”.*

**AR:**Thank you for pointing this out.We deleted the redundant "Fig 7" on line 298.

**RC***:[4]The figures are not clear.*

**AR:**Thank you.We have increased the resolution of each figure in the paper to 300-500dpi, and tested the quality of each figure in PACE. There is no ambiguity of the figure. However, after uploading figures and generating PDF files in the submission system, the resolution of each figures in the PDF file will be reduced. Please click the picture download link in the upper right corner of the PDF file to obtain high-resolution and high-quality figures.
